# 

**Published:** 1898-10-29

**Authors:** 


					THE HOSPITAL. " The standard of highest purity."?Lancet. Oat. 29th, 1898.
CADBURY'S MgA. o few CADBURY'S
rnnn. ?N| COCOA
isuuwm - to jm NO drugs or alkalies used.
ABSOLUTELY PURE, ^3 ?
'fiWICH HOSPISAJ.
No. 6311. m>XX?V. [S^] Saturday,
Oct. 29th, 1898.
[Snbsc?eP0tip.n78.ermS'] PRICE TWOPENCE.

				

